# Die Biomarker BNP und NT‑proBNP

**DOI:** 10.1007/s40664-022-00491-9

**Published:** 2023-01-16

**Authors:** G. M. Oremek, K. Passek, F. Holzgreve, V. von der Eltz, J. Dröge

**Affiliations:** 1 Abt. Labordiagnostik, Institut für Arbeitsmedizin, Sozialmedizin und Umweltmedizin, Goethe-Universität Frankfurt, Theodor Stern Kai 7, Haus 9a, 60590 Frankfurt am Main, Deutschland; 2 Institut für Arbeitsmedizin, Sozialmedizin und Umweltmedizin, Goethe-Universität Frankfurt, Frankfurt am Main, Deutschland

**Keywords:** Herzinsuffizienz, Arterielle Hypertonie, Koronare Herzkrankheiten, Immunologische Methoden, Diagnostik, Heart failure, Arterial hypertension, Coronary heart disease, Immunological methods, Diagnostics

## Abstract

Die vorliegende Übersicht über die Biomarker BNP und NT-pro-BNP wird im Rahmen der Serie „Biomarker“ des *Zentralblatts für Arbeitsmedizin, Arbeitsschutz und Ergonomie* publiziert, die sich mit dem immer häufigeren Gebrauch der Bestimmung von spezifischen Markern bei sog. Manager-Vorsorgen und Check-up-Untersuchungen beschäftigt. BNP und NT-pro-BNP eignen sich grundsätzlich als Marker zur Diagnosestellung der akuten und chronischen Herzinsuffizienz sowie zur Verlaufsbeurteilung. Hier zeigen diese eine hohe Sensitivität und Spezifität.

## Abstract (en)

The present review of the biomarkers BNP and NT-pro-BNP is published in the series “biomarkers” of the *Zentralblatt für Arbeitsmedizin, Arbeitsschutz und Ergonomie*, which deals with the increasing use of the determination of specific markers in so-called manager preventive and check-up examinations. In principle, BNP and NT-pro-BNP are fundamentally suitable as markers for diagnosing acute and chronic heart failure and for assessing the course. In this context these show a high sensitivity and specificity.

Die vorliegende Übersicht über den Biomarker BNP wird im Rahmen der Serie „Biomarker“ des *Zentralblatts für Arbeitsmedizin, Arbeitsschutz und Ergonomie* publiziert. Diese basiert auf Grundlagen der „Nationalen Versorgungs-Leitlinie Chronische Herzinsuffizienz“, den „2017 AAA/ACCF/HFSA Guideline for the Management of Heart Failure: A Report of the American College of Cardiology/American Heart Association Task Force on Clinical Practice Guidelines and the Heart Failure Society of America“, der „Richtlinie EU 2017/746 des Europäischen Parlaments“ und des „Rates über In-vitro-Diagnostika“ sowie der Neufassung der „Richtlinie der Bundesärztekammer zur Qualitätssicherung laboratoriumsmedizinischer Untersuchungen – Rili-BÄK“ [4, 19, 20, 37, 61, 62].

## Infobox Herzinsuffizienz (HI)

Herzinsuffizienz ist ein Syndrom, welches durch verschiedene Symptome (Kurzatmigkeit, Knöchelödeme und Fatigue) und Zeichen (erhöhte jugularvenöse Füllung, pathologische Atemgeräusche und periphere Ödemen) charakterisiert ist und ausgelöst wird durch strukturelle oder funktionelle kardiale Abnormalitäten [38, 53, 59, 61, 62]. Dies führt zu erhöhten intrakardialen Drücken oder einer reduzierten kardialen Auswurfleistung in Ruhe oder bei Belastung [38]. Die Herzinsuffizienz stellt eine führende und immer häufigere Ursache der Morbidität und Mortalität weltweit dar und wird als epidemisch mit einer geschätzten Prävalenz von über 37,7 Mio. Menschen beschrieben [38, 47, 51, 52, 59].

Es existieren einige Klassifikationssysteme, z. B. das der New York Heart Association oder die Leitlinien der American College of Cardiology (ACC) und American Heart Association (AHA; [38, 47, 61, 62, 64]). Dieses teilt die Patienten in folgende Gruppen ein:

- Stage A: Patienten mit dem Risiko einer sich entwickelnden Herzinsuffizienz
- Stage B: Patienten mit einer strukturellen Herzerkrankung ohne Symptome oder Anzeichen
- Stage C: Symptomatische Patienten
- Stage D: Patienten mit einer fortgeschrittenen Herzinsuffizienz [38, 53]

Die Prävalenz des Stadiums B ist 2‑ bis 3‑mal höher als die einer symptomatischen Herzinsuffizienz [1, 38, 39, 53].

Ein wichtiges praktisches Unterscheidungskriterium stellt die Einteilung in eine akute und chronische HI sowie in eine HI mit reduzierter linksventrikulärer (≤ 40 %) und erhaltener linksventrikulärer Funktion (≥ 50 %) dar [38, 53]. Etwa 10–20 % der Patienten weisen eine mittelgradige Ejektionsfraktion von 40–49 % auf [38].

*Chronische Herzinsuffizienz*

Die chronische HI betrifft etwa 2 % aller Erwachsenen weltweit, die Prävalenz ist altersabhängig und reicht von weniger als 2 % der Patienten unter 60 Jahren bis zu mehr als 10 % der Patienten über 75 Jahren [3, 11, 18, 27, 54]. Häufig bestehen Vorerkrankungen wie arterielle Hypertonie, koronare Herzkrankheit, Kardiomyopathien, Klappenerkrankungen oder eine Kombination dieser Krankheitsbilder [2, 18, 30, 38, 55].

Aufgrund einer immer älter werdenden Bevölkerung und einer verbesserten Therapie akuter kardialer Ereignisse geht man von einem Prävalenzanstieg von 25 % in den nächsten 20 Jahren aus [44–46, 57, 59]. Die Therapiekosten machen einen Anteil von 2–3 % der gesamten Ausgaben des Gesundheitssystems von Staaten mit hohem Einkommen aus und werden nach Berechnungen in den nächsten 20 Jahren um 200 % ansteigen [60, 61, 64].

Patienten mit HI zeichnen sich durch eine schlechte Prognose mit hohen Hospitalisationsraten und einer hohen Mortalität aus [38].

Die Pathophysiologie der HI mit reduzierter Ejektionsfraktion ist durch fortscheitende Ereignisse geprägt: Risikofaktoren führen zu kardialen Schäden und anschließend zur Entwicklung einer initial asymptomatischen myokardialen Dysfunktion sowie schließlich zu einer Verschlimmerung der Symptome bis zur endgradigen Herzinsuffizienz [13, 34–36]. Diese Risikofaktoren beinhalten die koronare Herzkrankheit, die arterielle Hypertonie, Hypercholesterinämie, Diabetes mellitus, Fettleibigkeit, eine positive Familienanamnese, eine Prädisposition für Kardiomyopathien und die Exposition gegenüber kardiotoxischen Substanzen wie Alkohol, Amphetaminen, Krebsmedikamenten und radioaktiver Strahlung [12, 33, 37, 38, 40, 41, 43, 50, 53]. Der Verlust von Myozyten und eine erhöhte Belastung führen zu einer exzentrischen Hypertrophie der verbleibenden Muskelzellen sowohl direkt als auch durch eine neurohumorale Aktivierung sowie zu einer Fibrosierung, einer progressiven linksventrikulären Dilatation, einer Veränderung der Form des linken Ventrikels von elliptisch zu kugelförmig und häufig zu einer Mitralklappeninsuffizienz [25, 26, 31, 32, 42, 54, 56, 58, 61]. Diese Umbauvorgänge, auch als linksventrikuläres Remodeling bezeichnet, führen zu einem erhöhten myokardialen Sauerstoffverbrauch und zu einer reduzierten Effizienz der myokardialen Kontraktion [11, 38, 44, 48, 49, 60, 65]. Die neurohumorale Aktivierung sorgt für eine renale Natriumretention, eine Volumenüberladung und Ödeme [9]. Eine gleichzeitige renale Funktionsstörung verursacht ein reduziertes Ansprechen auf Diuretika sowie eine verschlechterte Prognose [21–24, 28]. Eine Stauung des Darms führt zur Kachexie, zur Aktivierung von Inflammation und schließlich zu einer weiteren Verschlechterung der Prognose [12, 14, 17].

Die Pathophysiologie der Herzinsuffizienz mit erhaltener Ejektionsfraktion ist noch nicht vollständig verstanden: Eine Hypothese besagt, dass Komorbiditäten wie z. B. Adipositas, eine chronische Nierenerkrankung, Eisenmangel, Hypertonie, Diabetes mellitus und die chronisch-obstruktive Lungenerkrankung einen Zustand der systemischen Proinflammation hervorrufen mit Produktion reaktiver Sauerstoffspezies durch das Koronarendothel, reduzierter Bioverfügbarkeit von NO und erniedrigter Proteinkinase-G-Aktivität [9]. Dieser Status führt zur Myozytenhypertrophie, Titin-Hypophosphorylation, Kollagenablagerung und einer herabgesetzten linksventrikulären Compliance [9, 24, 25].

Die typischen Symptome einer HI umfassen Kurzatmigkeit, Orthopnoe, paroxysmale nächtliche Dyspnoe, Fatigue, Palpitationen, Knöchelschwellung, schnell eintretendes Sättigungsgefühl und Blähungen, Anorexie, Depression, Verwirrtheit, Kachexie und die Zeichen eines erhöhten jugularvenösen Drucks, einen verschobenen Herzspitzenstoß, pathologische Herzgeräusche, einen dritten Herzton, Lungengeräusche, Pleuraergüsse, Hepatomegalie, hepatojugulären Reflux, Aszites und periphere Ödeme [9, 10, 12, 17, 52]. Aufgrund der geringen Sensitivität und Spezifität der klinischen Symptome und Zeichen stellt die Messung der Plasmakonzentration des „brain natriuretic peptide“ (BNP) oder des N‑terminalen Prohormons des BNP (NT-pro-BNP) einen Hauptpfeiler der Diagnostik der Herzinsuffizienz mit exzellenter Sensitivität und negativem prädiktivem Wert dar [5–8, 13, 15, 16].

Eine Echokardiographie alleine ist nicht ausreichend zur Diagnosestellung einer HI, liefert allerdings trotzdem wichtige Informationen über die Anatomie, wie strukturelle Abnormalitäten, linksventrikuläre Hypertrophie, Vergrößerung des linken Vorhofs, Zeichen der diastolischen Dysfunktion, Klappenfehler, Einschränkungen der rechtsventrikulären Funktion, und sie kann den pulmonalarteriellen Druck schätzen [9, 17, 28–30].

Eine reduzierte Ejektionsfraktion ist mit einer erhöhten Gesamtmortalität, mit dem Tod aufgrund einer Herzerkrankung und einer erhöhten Hospitalisierungsrate aufgrund von Herzinsuffizienz vergesellschaftet [9, 27, 28].

Eine Kardio-MRT weist eine bessere Genauigkeit und Reproduzierbarkeit als die Echokardiographie auf mit einer besseren Gewebecharakterisierung und räumlicher Auflösung, was bei der Diagnosestellung eines entzündlichen oder infiltrativen Prozesses helfen kann [9]. Allerdings ist der Gebrauch limitiert durch hohe Kosten und eine Inkompatibilität mit Defibrillatoren und Herzschrittmachern [9]. Die Angiographie ist indiziert bei Patienten mit Angina und einer mittleren bis hohen Vortestwahrscheinlichkeit für eine koronare Herzkrankheit (KHK) und bei denjenigen, welche für eine Revaskularisation geeignet sind [9]. Eine Kardio-CT kann zum Ausschluss einer KHK bei Patientin mit einer niedrigen bis mittleren Vortestwahrscheinlichkeit diskutiert werden [9, 10].

Die Prävention der Herzinsuffizienz sollte ein primäres Ziel des Gesundheitssystems sein und beinhaltet die Prävention und Therapie der arteriellen Hypertonie und der modifizierbaren Risikofaktoren der KHK [9, 10]. So reduziert eine Senkung des systolischen Blutdrucks auf Werte unter 120 mm Hg bei Hochrisikopatienten im Vergleich zu Werten unter 140 mm Hg die Mortalität, das Auftreten kardiovaskulärer Ereignisse und die Hospitalitationsraten [9, 31, 32]. Patienten mit kardiovaskulären Risikofaktoren profitierten von einem BNP-Screening und einer Therapie bei erhöhten Konzentrationen mit Renin-Angiotensin-Aldosteron-System-Antagonisten bezüglich einer Reduzierung der Raten einer asymptomatischen linksventrikulären Dysfunktion oder symptomatischen Herzinsuffizienz [9, 33].

Den Antidiabetika Liraglutide, ein Glucagon-like Peptid-1-Agonist und Empaglifozin, ein Natrium-Glukose-Cotransporter-2-Inhibitor wird teilweise eine positive Wirkung in Bezug auf kardiale Ereignisse zugeschrieben [9, 34–37].

Auch ist es möglich, Überwachungsgeräte zu implantieren, die den rechtsventrikulären, linksatrialen oder pulmonalarteriellen Druck messen und somit frühzeitig eine Verschlechterung der HI anzeigen [9, 38].

Die Therapie der HI mit reduzierter Ejektionsfraktion stützt sich sowohl auf die Einnahme von Diuretika, um Stauungssymptome zu reduzieren, als auch auf neurohumorale Antagonisten und die Implantation von Kardioverter-Defibrillatoren, um das Outcome zu verbessern [9, 10, 39, 40]. Zu den letztgenannten zählen ACE-Hemmer, Angiotensin-Rezeptor-Blocker, Beta-Blocker, Mineralokortikoid-Rezeptor-Antagonisten (MRA) sowie Angiotensin-Rezeptor-Blocker Neprilysin-Inhibitoren [9, 10, 39, 40]. Eine optimale Therapie erfordert häufig einen multidisziplinären Ansatz mit einer adäquaten Patienten- und Ernährungsschulung, Erkennen von frühen Symptomen und sportlicher Betätigung [9, 39].

Bei fortgeschrittener HI mit weiterhin trotz Therapie bestehender schwerer Symptomatik ist bei fehlender Kontraindikation durch Alter oder schwerer anderer Komorbiditäten die Herztransplantation indiziert [9, 41, 42].

Keine Therapie hat einen definitiven positiven Effekt auf die primären Endpunkte in Outcome-Untersuchungen der HI mit erhaltener Ejektionsfraktion [9]. Für Patienten mit Stauungssymptomatik eignen sich ebenfalls Diuretika, wobei sorgfältig eine zu starke Senkung der linksventrikulären Vorlast vermieden werden sollte. Komorbiditäten wie Hypertonie, KHK und Vorhofflimmern sollten behandelt werden [9, 10, 39]. Der Einsatz von Spironolacton hatte zwar keinen Effekt auf die Mortalität oder die Symptome, reduzierte aber in einigen Studien die Hospitalisierungsrate [9, 39, 43, 44].

*Akute Herzinsuffizienz*

Die akute Herzinsuffizienz, welche eine steigende Prävalenz zeigt, wird definiert als neu aufgetretene oder wiederkehrende Symptome und Zeichen einer HI und erfordert eine umgehende Diagnostik und Therapie [9, 39, 45]. In Ländern mit hohem Einkommen stellt die akute HI die häufigste Ursache für eine Hospitalisation von Patienten über 65 Jahren dar [9]. Etwa 3 Mio. Personen werden jährlich mit der primären oder sekundären Diagnose einer HI in ein Krankenhaus eingewiesen, wobei die akute HI mehr als 7 Mio. Krankenhaustage jährlich alleine in den USA verursacht [9, 16]. Die Krankenhausmortalität der akuten HI kann größer sein als die für einen akuten Myokardinfarkt und liegt zwischen 2 und 20 %, wobei die Mortalität für über ein Jahr nach Entlassung deutlich höher liegt als für Patienten mit einer chronischen HI [9, 46, 47].

Die akute HI ist ein heterogenes Syndrom mit vielen Parallelen zur chronischen HI, wobei die Prognose eines Patienten mit chronischer HI nach einer akuten Episode deutlich schlechter ist [9, 46]. Symptome oder Zeichen einer reduzierten Perfusion finden sich lediglich in 5 %, wobei eine stationäre Aufnahme hauptsächlich aufgrund von Stauungssymptomen wie Dyspnoe und peripheren Ödemen erfolgt [9]. Viele Faktoren spielen hierbei eine entscheidende Rolle, wie die ventrikuläre diastolische Funktion, der pumonalarterielle Druck und die Resistance, eine reduzierte Flüssigkeitsausscheidung der Lungen und der peripheren Gewebe, sowie der intravasale Volumenstatus [9]. Eine geringe kardiale Auswurfleistung, eine alterierte intrarenale Hämodynamik, der tubuloglomeruläre Feedback-Mechanismus und ein erhöhter intraabdomineller Druck oder ein erhöhter venöser Druck können eine Verschlechterung der Nierenfunktion auslösen [9, 22, 48, 49]. Zudem kommt es zur exzessiven Ausschüttung von Neurohormonen und Zytokinen, die durch oxidativen Stress eine Inflammation auslösen [9].

Die Diagnosestellung erfolgt u. a. durch die körperliche Untersuchung, EKG, röntgenologische Untersuchung des Thorax, Lungensonographie, Herzechokardiographie und durch die Bestimmung der natriuretischen Peptide BNP und NT-proBNP, welche eine hervorragende Sensitivität bei der Diagnosestellung der akuten HI aufweisen [9, 10, 39]. Aufgrund der geringeren Spezifität müssen diese auch im Kontext anderer Erkrankungen wie der pulmonalarteriellen Embolie, Rechtsherzbelastung oder Myokardinfarkt und Patientencharakteristika wie Alter, Geschlecht, Nierenfunktion oder Adipositas betrachtet werden [9, 60].

Die Ziele der Therapie stellen die unmittelbare Stabilisation und die Symptomkontrolle dar, wobei besonders Diuretika und Vasodilatatoren eingesetzt werden [9, 10, 50]. Eine bereits bestehende Therapie mit Betablockern sollte fortgeführt werden sowie die Einnahme von MRAs oder ACE-Hemmern, wenn keine schwere renale Dysfunktion oder Hyperkaliämie besteht [9, 51].

Im Allgemeinen kann eine HI durch viele verschiedene Pathologien hervorgerufen werden, wobei zu zwei Dritteln ischämische Herzkrankheiten, eine chronisch-obstruktive Lungenerkrankung, eine hypertensive Herzerkrankung oder rheumatische Herzerkrankungen ursächlich sind [52]. Des Weiteren sind koronare Dissektionen, koronare Embolien, valvuläre Erkrankungen, genetische Fehlbildungen, primäre Kardiomyopathien, wie hypertrophe oder arrhythmogene Kardiomyopathien, mitochondriale Myopathien, Ionenkanalpathologien, stressinduzierte oder infektiologisch induzierte Funktionsstörungen möglich [52]. Sekundäre Kardiomyopathien umfassen z. B. Amyloidose, Sarkoidose, Speicherkrankheiten, Sklerodermie [52]. Auch Alkohol- sowie Nikotinabusus, der Konsum von Kokain oder bestimmte Toxine wie Anthrazykline, aber auch Schlafentzug können zum Auftreten einer Herzinsuffizienz beitragen [52, 53]. Die Exposition gegenüber Feinstaub kann die Morbidität und Mortalität kardiovaskulärer Erkrankungen erhöhen, genauso wie Ozon, Kohlenstoffmonoxid, Nitrogendioxid, Schwefeldioxid sowie flüchtige organische Verbindungen [54].

## Biochemie von B-Typ-natriuretisches Peptid (BNP) und aminoterminales proBNP (NT-pro-BNP)

Das Peptid BNP wird in Kardiomyozyten bei Wandstress als 134 Aminosäuren langes Prä-proBNP synthetisiert und vom kurzen Arm des Chromosoms 1 kodiert, wobei posttranslational nach Abspaltung eines Signalpeptids zunächst pro-BNP entsteht [55, 58–60]. Das proBNP wird durch die Serin-Protease Corin in BNP mit einem Molekulargewicht von 3,5 kDa und in das biologisch inaktive NT-pro-BNP mit einem Molekulargewicht von 8,5 kDa gespalten [55, 56, 58, 59]. Die Halbwertszeit von BNP beträgt etwa 20 min, die von NT-proBNP 1–2 h [55, 58–60]. Zu den Wirkungen von BNP zählen eine sympathomimetische Aktivität im zentralen und peripheren Nervensystem, Hemmung des Trinkens, Natriurese und Diurese, Hemmung des Renin-Angiotensin-Aldosteron-Systems, Relaxation der glatten Muskulatur und Vasodilatation, Erhöhung der Permeabilität des Gefäßendothels, Relaxation der glatten Muskulatur der tiefen Atemwege, Steigerung der Lipolyse im Fettgewebe sowie Hemmung des kardialen und vaskulären Remodelings [3, 6, 10, 55, 56, 58, 60]. Bei linksventrikulärer Fehlfunktion, Ventrikelhypertrophie und anderen kardialen Dysfunktionen mit chronisch erhöhtem hämodynamischem Druck oder Volumenüberladung unterliegen die ventrikulären Myozyten Modifikationen und reexprimieren fetale Gene, die eine verstärkte Synthese von BNP kodieren [55, 59].

Beide Peptide werden durch glomeruläre Filtration eliminiert, BNP zusätzlich noch durch den BNP-Clearance-Rezeptor, sodass bei Patienten mit Niereninsuffizienz NT-proBNP stärker erhöht ist als BNP [5, 55, 59, 60].

BNP und NT-pro-BNP sind bei HI erhöht, und ihre Konzentration im Blut steigt mit dem Ausmaß und der Dauer der ventrikulären Dysfunktion des Herzens an [5, 6, 55, 56, 58–60].

Die Konzentrationen sind alters- und geschlechtsabhängig und die Tests nicht standardisiert, sodass herstellerspezifische Angaben zu den oberen Referenzwerten beachtet werden müssen [5, 55, 56, 59]. Diese steigen mit dem Alter an und werden bei Frauen höher angegeben als bei Männern [55, 59]. Dunkelhäutige Menschen weisen niedrigere Konzentrationen auf [5, 59, 62]. Die Bestimmung sollte in Zusammenhang mit der Anamnese, dem klinischen Bild und anderen Untersuchungen, wie z. B. dem Echokardiogramm, erfolgen [55, 60]. NT-pro-BNP hat bei Patienten, die sich mit Luftnot und Leistungsinsuffizienz vorstellen, eine diagnostische Sensitivität von 88 % bei einer Spezifität von 92 %, einen positiven prädiktiven Wert von 96,7 % und einen negativen von 80,6 % [55, 57]. Die Werte von BNP und NT-pro-BNP korrelieren mit der Einteilung der HI nach der New York Heart-Association(NYHA)-Klasse [2, 55, 59].

Patienten mit akuter Dyspnoe und akuter Stauungsinsuffizienz des Herzens weisen in der Regel höhere Konzentrationen als diejenigen mit nichtakuter HI auf [55, 59].

Beide Herzmarker steigen mit Abnahme der glomerulären Filtrationsrate an und beeinflussen so die Entscheidungsgrenze, zudem haben adipöse Patienten niedrigere Werte [7, 8, 55, 59, 60].

BNP und NT-pro-BNP sind bei chronischer HI bessere prognostische Indikatoren als die NYHA-Klassifikation und bei stabiler Angina pectoris geben BNP und NT-proBNP Hinweise zu längerfristig auftretenden kardiovaskulären Ereignissen und zur Mortalität [55, 59]. Bei Patienten mit kürzlich erfolgtem Myokardinfarkt geben beide Marker Auskunft zur linksventrikulären Funktion, zur Infarktgröße und zum Überleben, außerdem zur Prognose bei Volumenüberladung beim akuten Koronarsyndrom, Vorhofflimmern und der Lungenembolie [51–54, 58, 60]. Bei Patienten mit einer prognostischen Überlebenszeit von 2–4 Jahren konnte in einer Studie eine 3,5 bis 5,6-fach höhere BNP-Konzentration als bei längerzeitig Überlebenden nachgewiesen werden [5, 43, 55, 59].

BNP und NT-proBNP haben eine geringere diagnostische Sensitivität und Spezifität, wenn es sich um eine diastolische Dysfunktion handelt [55].

Bei akut dekompensierter HI sollte es durch eine intensivierte Rekompensation zu einem mindestens 30 %igen Abfall von NT-pro-BNP kommen [5, 55].

Bei Patienten mit akutem Koronarsyndrom sollten Konzentrationsbestimmungen bei Aufnahme und 2–5 Tage nach Eintritt der Symptome durchgeführt werden, wobei es bei einem STEMI zu einem raschen Anstieg von BNP und stärker von NT-proBNP mit Gipfelwerten innerhalb von 12–24 h und einer Gipfelkonzentration, die proportional zur Größe des Infarkts ist, kommt [5, 55, 59]. Die Biomarkerwerte nehmen anschließend kontinuierlich ab, bleiben aber bis zu 12 Wochen erhöht [55]. Bei einigen Patienten, insbesondere denjenigen mit ausgedehntem Infarkt, kommt es am 5. Tag zu einem zweiten Gipfel, der wahrscheinlich einen negativen Verlauf im Remodeling des Myokards anzeigt [11, 55].

Bei akuter Lungenembolie ist die Bestimmung von BNP und NT-proBNP zusätzlich zur Echokardiographie eine Hilfe zur Diagnostik einer rechtsventrikulären HI und ein Prognosemarker der intrahospitalen Mortalität [5, 55, 59].

Die periphere arterielle Verschlusskrankheit zeigt mit erhöhten NT-proBNP-Werten ein ansteigendes Mortalitätsrisiko [55, 65].

Die Bestimmung von BNP und kardialem Troponin vor und 20 h nach einer Herzoperation geben Auskunft über eine sich entwickelnde kardiale Dysfunktion: Patienten mit einem postoperativen Anstieg beider Marker hatten ein 12-fach erhöhtes Risiko der HI [5, 55].

Beim Vorliegen einer COVID-19-Infektion bei Patienten mit vorbestehender kardialer Erkrankung sind natriuretische Peptide Prognoseparameter für eine mögliche intensivmedizinische Überwachung, Beatmung und Tod [61, 62].

Die Höhe der Werte von BNP und NT-pro-BNP sind vom Typ des verwendeten Assays, der Antikörperspezifität und dem Kalibriermaterial abhängig [55, 56, 58]. Zur Bestimmung von BNP mittels Immunoassay sollte die Messung innerhalb von 4 h aus EDTA-Blut bei Raumtemperatur erfolgen. Ist die Messung in dieser Zeit nicht möglich, so sollte die Trennung von den korpuskulären Bestandteilen erfolgen und das Plasma in einem Röhrchen, das einen Kallikrein- oder Serin-Protease-spezifischen Inhibitor enthält, bei 4 °C bis zu 72 h gelagert werden, wobei alternativ das Tieffrieren möglich ist [55, 56]. NT-proBNP ist im Vollblut oder Plasma bei Raumtemperatur oder 4 °C bis 72 h stabil [54–56].

Verschiedene Einflussgrößen sollten beachtet werden: Die Blutentnahme sollte nicht nach körperlich belastenden Untersuchungen wie Ergometrie oder Stress-Echokardiographie erfolgen, da unter solchen Bedingungen auch bei Gesunden Konzentrationserhöhungen auftreten können [55, 63]. Medikamente wie Diuretika, ACE-Hemmer und Betablocker können die BNP-Konzentration im Plasma vermindern, außerdem haben adipöse Patienten niedrigere Werte, eine Kochsalzbelastung führt zu einer Erhöhung [55, 60].

Weitere Ursachen für eine Konzentrationserhöhung der natriuretischen Peptide sind eine Myokarditis, eine Kardioversion, eine toxische Myokardschädigung z. B. durch Chemotherapeutika, Anämie, obstruktive Schlafapnoe, schwere Pneumonie, kritische Krankheitszustände, bakterielle Sepsis und schwere Verbrennungen [5].

## Indikation von BNP und NT-proBNP

Unter folgenden Bedingungen ist die Indikation der BNP und NT-proBNP-Bestimmung gegeben:

- Diagnose oder Ausschluss einer Herzinsuffizienz, z. B. bei akut aufgetretener Dyspnoe,
- Abschätzung der Prognose einer Herzinsuffizienz, z. B. bei akutem Koronarsyndrom,
- Beurteilung des Schweregrades einer Herzinsuffizienz nach NYHA bzw. des American College of Cardiology/American Heart Association,
- Risikostratifizierung bei Lungenembolie,
- therapeutisches Monitoring der Herzinsuffizienz.

Aufgrund geringer Organ- und Tumorspezifität sowie eines geringen positiven prädiktiven Werts sind Biomarker zum Screening asymptomatischer Patienten in Check-up-Untersuchungen generell ungeeignet [1, 5, 7, 9, 55–57, 59, 60, 64, 65].

## Ausblick

Werden die unterschiedlichsten Aspekte der arbeitsmedizinischen Vorsorge, wie sie aktuell im *Zentralblatt für Arbeitsmedizin, Arbeitsschutz und Ergonomie* publiziert und diskutiert werden, betrachtet, so wird klar, dass BNP und NT-proBNP-Bestimmungen keine Berechtigung im Rahmen von iGeL-Leistungen bzw. Managerchecks im Kontext der Arbeitswelt haben, wohl aber im Bereich der Kardiologie.

## Fazit für die Praxis

- Die Biomarker BNP und NT-proBNP eignen sich zur Diagnosestellung der akuten und chronischen Herzinsuffizienz sowie zur Verlaufsbeurteilung.
- Es besteht in der Regel eine gute Korrelation der Werte zum klinischen Verlauf unter der Herzinsuffizienztherapie. Erhöhte Konzentrationen vor einer Behandlung deuten auf eine schlechte Prognose hin.
- In der Primärdiagnostik ist die Bestimmung von BNP und NT-proBNP aufgrund einer zu geringen diagnostischen Sensitivität nicht einsetzbar, daher sind diese Biomarker nicht unkritisch im Rahmen von Screenings oder „Check-up“-Untersuchungen zu verwenden.
- Die Konzentrationen weichen bei vielen verschiedenen Krankheiten, wie u. a. der Niereninsuffizienz, Vorhofflimmern, Lungenembolie, der peripheren arteriellen Verschlusskrankheit, Myokarditis, einer toxische Myokardschädigung, z. B. durch Chemotherapeutika, Anämie, obstruktive Schlafapnoe, schwere Pneumonie, kritische Krankheitszustände, bakterielle Sepsis und schwere Verbrennungen, aber auch bei der Einnahme von verschiedenen Medikamenten vom Referenzwert ab.
- Die BNP und NT-proBNP-Konzentrationsbestimmung wird beim Verdacht auf eine Herzinsuffizienz eingesetzt und dient hier auch dem therapeutischen Monitoring und der Prognoseabschätzung.
- Analog zu anderen Biomarkern ist die Bestimmung von BNP und NT-proBNP aufgrund der zu geringen Spezifität und Sensitivität nicht als Screeninguntersuchung geeignet, sondern sollte nur bei klinischem Verdacht auf eine Herzinsuffizienz in Verbindung mit anderen Untersuchungen angewendet werden.
